# Natural chemotherapeutic alternatives for controlling of haemonchosis in sheep

**DOI:** 10.1186/s12917-019-2050-2

**Published:** 2019-08-20

**Authors:** Dominika Mravčáková, Zora Váradyová, Anna Kopčáková, Klaudia Čobanová, Ľubomíra Grešáková, Svetlana Kišidayová, Michal Babják, Michaela Urda Dolinská, Emília Dvorožňáková, Alžbeta Königová, Jaroslav Vadlejch, Adam Cieslak, Sylwester Ślusarczyk, Marián Várady

**Affiliations:** 1 Centre of Biosciences, Institute of Animal Physiology, Slovak Academy of Sciences, Košice, Slovak Republic; 2 Institute of Parasitology, Slovak Academy of Sciences, Hlinkova 3, 040 01 Košice, Slovak Republic; 3 Department of Zoology and Fisheries, Czech University of Life Sciences Prague, Faculty of Agrobiology, Food and Natural Resources, Suchdol, Prague Czech Republic; 4 Department of Animal Nutrition, Poznan University of Life Sciences, Poznan, Poland; 5 Department of Pharmaceutical Biology with Botanical Garden of Medicinal Plants, Medical University of Wroclaw, Wroclaw, Poland

**Keywords:** 16S rRNA gene, Total antioxidant capacity, *Haemonchus contortus* antigen, Phytochemicals, UHRMS

## Abstract

**Background:**

Parallel in vitro and in vivo experiments were designed to evaluate promising chemotherapeutic alternatives for controlling haemonchosis in ruminants. In vitro anthelmintic activities (egg hatch test – EHT; larval development test – LDT) of aqueous and methanolic herbal extracts Mix1 and Mix2 were investigated. The in vivo effects of dietary supplementation with Mix1 and Mix2 on the parasitological status, inflammatory response, antioxidant parameters and microbial community of the lambs infected experimentally with *Haemonchus contortus* were investigated*.* Lambs were divided into four groups for the in vivo study: uninfected control lambs (C), infected lambs (I), infected lambs supplemented with Mix1 (I + Mix1) and infected lambs supplemented with Mix2 (I + Mix2). The experimental period was 70 days.

**Results:**

The number of eggs per gram (EPG) of feces was quantified 22, 30, 37, 44, 51, 58, 65 and 70 days post-infection, and mean abomasal worm counts were assessed 70 days post-infection. Quantitative analyses identified 57.3 and 22.2 mg/g phenolic acids, 41.5 and 29.5 mg/g flavonoids and 1.4 and 1.33 mg/g protoberberine-type alkaloids in Mix1 and Mix2, respectively. The methanolic extracts of the herbal mixtures in both in vitro tests had higher anthelmintic effects (*P <* 0.01) than the aqueous extracts, but the effects did not differ significantly between Mix1 and Mix2 (*P >* 0.05). I + Mix1 and I + Mix2 lowered mean EPGs between 44 and 70 d by 58.1 and 51.6%, respectively. The level of IgG antibodies against *H. contortus* increased significantly after infection in each infected group.

**Conclusion:**

These results represent the first monitoring of the in vitro anthelmintic effects of herbal mixtures on *H. contortus*. The in vivo experiment indicated that the anthelmintic effect was not sufficient for the elimination of parasites, but this herbal treatment may affect the host over a longer term, reducing the parasitic infection in the host.

## Background

Modern production systems require the use of nutraceuticals for optimal production and health in ruminant nutrition. Gastrointestinal nematode (GIN) infections are the prevalent parasitic diseases contributing to the morbidity and mortality of various livestock species worldwide. Infection by the GIN *Haemonchus contortus* is mainly controlled by chemoprophylaxis by the repeated application of anthelmintics, which has led to the increased risk of anthelmintic resistance and multidrug resistance [1, 2]. Resistance to anthelmintics also applies to the novel anthelmintic monepantel [3, 4]. Herbs with important biological activities are potential nutraceuticals for controlling GINs in ruminants [5–7]. A variety of herbs from traditional medicine with health-promoting properties have been used to treat various diseases in both humans and animals for centuries [8]. Alternative control approaches can therefore involve combinations of traditional herbal medicines [9, 10], the pharmacologically active plant compounds [11, 12] and the self-medication of grazing animals [13, 14]. Polyphenols, especially tannins, flavonoids and phenylpropanoids, mainly have anthelmintic [15, 16] and antioxidative and anti-inflammatory [17, 18] properties. Hoste et al. [19] have demonstrated that the manipulation of host nutrition provides useful options for controlling GINs as a component of an integrated multidisciplinary strategy. The ability of the host to resist GIN infections also depends on the development of a protective acquired immune response [20]. Interactions between intestinal parasites, microbial communities and immune systems play a relevant role in modulating each other and in the maintenance of homeostasis [21].

Our previous trials found that the dry medicinal herbs can be used as chemotherapeutic alternatives for controlling of haemonchosis in ruminants based on their beneficial effect [22–24]. The bioactive phytochemicals responsible for anthelmintic, antioxidant and anti-inflammatory activities in various herbal mixtures have not yet been characterized in vitro. Our goal was therefore to examine (1) the in vitro anthelmintic activities of extracts from two dry mixtures of medicinal herbs against *H. contortus*, and (2) the in vivo impact of these mixtures on inflammatory, parasitological, antioxidant, mineral and microbial community parameters of lambs experimentally infected with *H. contortus.*

## Results

### Bioactive compounds

The herbal compositions (Table 1), fermentation parameters (Table 2) and bioactive compounds (Tables 3, 4) of Mix1 and Mix2 are presented. Quantitative analyses of Mix1 identified 57.3 mg/g phenolic acids (Nos. 1–9, 19, 21, 26 and 28) and 41.5 mg/g flavonoids (Nos. 10–18, 20, 22–25, 27 and 29–33) (Table 3). Quantitative analyses of Mix2 identified 22.2 mg/g phenolic acids (Nos. 1–6, 20, 23, 26 and 29) and 29.5 mg/g flavonoids (Nos. 7–19, 21, 22, 24, 25, 27, 28 and 30) (Table 4). Protoberberine-type alkaloids were also identified in Mix1 (1.4 mg/g) and Mix2 (1.33 mg/g) based on comparisons with chelidonine and protopine standards.

**Table 1:** Compositions of the herbal mixtures

Mix1				Mix2			
Species	Family	Part used	% in Mix1	Species	Family	Part used	% in Mix2
*Artemisia absinthium* L.	Asteraceae	Stem	1.0	*Artemisia absinthium* L.	Asteraceae	Stem	1.0
*Matricaria chamomilla* L.	Asteraceae	Flower	13.4	*Achillea milefolium* L.	Asteraceae	Stem	12.4
*Foeniculum vulgare* Mill.	Apiaceae	Seed	5.0	*Calendula officinalis* L.	Asteraceae	Flower	12.4
*Fumaria officinalis* L.	Papaveraceae	Stem	13.4	*Matricaria chamomilla* L.	Asteraceae	Flower	12.4
*Hyssopus officinalis* L.	Lamiaceae	Stem	13.4	*Cichorium intybus* L.	Asteraceae	Stem	12.4
*Malva sylvestris* L.	Malvaceae	Flower	13.4	*Fumaria officinalis* L.	Papaveraceae	Flower	12.4
*Melissa officinalis* L.	Lamiaceae	Stem	13.4	*Hypericum perforatum* L.	Hypericaceae	Stem	12.4
*Plantago lanceolata* L.	Plantaginaceae	Leaf	13.4	*Malva sylvestris* L.	Malvaceae	Stem	12.4
*Solidago virgaurea* L.	Asteraceae	Stem	13.4	*Urtica dioica* L.	Urticaceae	Stem	12.4

Medicinal herbs (AGROKARPATY, Plavnica, Slovak Republic and BYLINY Mikeš s.r.o., Číčenice, Czech Republic)

**Table 2:** Fermentation parameters of the dietary substrates after 24 h of in vitro incubation

Parameter	Rumen fluid inoculum				Fresh fecal inoculum			
	Mix1	Mix2	MH	Concentrate	Mix1	Mix2	MH	Concentrate
pH	6.86	7.04	7.16	6.92	7.28	7.32	7.41	7.13
NH_3_-N (mg/L)	130	147	122	126	158	218	167	127
Total gas (mL/g DM)	180	178	120	222	91	90	61	140
CH_4_ (mmol)	6.99	6.68	7.90	7.62	6.49	6.21	7.05	7.06
Total SCFA (mmol/L)	48.3	47.8	42.4	53.4	46.8	47.2	43.1	49.3
Acetate (mol%)	60.0	60.3	69.1	63.4	66.3	65.9	66.1	67.2
Propionate (mol%)	16.4	16.4	17.2	19.1	20.1	20.9	20.2	23.7
Butyrate (mol%)	10.9	10.3	10.4	13.1	3.14	2.82	2.50	8.82
Total ciliate protozoa (10^3^/mL)	94	99	114	160	–	–	–	–
*Entodinium* spp. (10^3^/mL)	91	96	111	155	–	–	–	–
*Epidinium* spp. (10^3^/mL)	2.96	3.19	3.17	5.39	–	–	–	–

*Mix1* herbal mixture 1, *Mix2* herbal mixture 2, *Concentrate* commercial concentrate composed of barley, soybean meal, wheat bran, bicarbonate and mineral–vitamin premix, *MH* meadow hay

**Table 3:** Contents of the main bioactive compounds identified in the Mix1 analyzed in negative-ionization mode

No.	Compound	RT (min)	UV	*m/z* [M-H]^−^	Formula	MS^2^ main ion	MS^2^ fragments	mg/g DM
1	4-O-Caffeoylquinic acid	3.8	215/325	353.0880	C_16_H_18_O_9_	191.0553		0.71
2	3-O-Caffeoylquinic acid	5.2	215/325	353.0880	C_16_H_18_O_9_	191.0556	179/161/135	11.3
3	5-O-Caffeoylquinic acid	5.5	215/325	353.0880	C_16_H_18_O_9_	191.0557		0.73
4	Caffeic acid	6.9	215/324	179.0339	C_9_H_8_O_4_	135.0435		0.81
5	1-O-Feruloylglucose	8.0	215/324	355.1041	C_16_H_20_O_9_	193.0498	149/0589/134	0.38
6	3-O-p-Coumaroylquinic acid	8.7	215/325	337.0933	C_16_H_18_O_8_	191.0187	173	0.65
7	4-O-p-Coumaroylquinic acid	9.5	215/325	337.0933	C_16_H_18_O_8_	191.0187	173	3.45
8	Caffeoylmalic acid	9.7	313/000	295.0455	C_13_H_12_O_8_	179.0336	133/207	1.42
9	4-Caffeoylshikimic acid	11.1	215/325	335.0768	C_16_H_16_O_8_	179.0334	161/191/135	6.40
10	Glucodistylin	11.5	218/295	465.1024	C_21_H_22_O_12_	285.0405	303/177	4.90
11	Myricetin 3-O-galactosid	12.2	271/345	479.0828	C_21_H_20_O_13_	317.0297		20.2
12	Quercetin O-Pen-Hex	12.3	252/351	595.1298	C_26_H_28_O_16_	300.0273		2.63
13	Quercetin O-Hex-O-Dhex	12.4	252/351	609.1442	C_27_H_30_O_16_	447.1131	152/429/161/179	0.96
14	Luteolin O-diglucuronide	12.5	255/340	637.1029	C_27_H_26_O_18_	461.0707	285/04	0.26
15	Rutin	13.5	255/342	609.1451	C_27_H_30_O_16_	300.0275		2.96
16	Acetoside (verbascoside)	13.9	218/291/331	623.1972	C_29_H_36_O_15_	461.1657	161/153/179/135	0.63
17	Quercetin O-Hex	15.0	257/351	463.0874	C_21_H_20_O_12_	300.0273		0.02
18	Quercetagetin 3′-methyl-ether 7-glucoside	15.1	252/351	493.0977	C_22_H_22_O_13_	447.0928	331/285	0.30
19	3,5-Dicaffeoylquinic acid	15.2	215/325	515.1195	C_25_H_24_O_12_	353.0877	173/179/335/191/161	0.42
20	Quercetin O-Pen	15.3	252/351	433.0776	C_20_H_18_O_11_	300.0277	271/255/151	1.41
21	1,5-Dicaffeoylquinic acid	15.4	215/325	515.1194	C_25_H_24_O_12_	353.0885	191/179/135	15.4
22	Qquercitrin	15.4	256/348	447.0904	C_21_H_20_O_11_	300.0276		1.88
23	Apigenin O-Hex	15.5	267/341	431.0983	C_21_H_20_O_10_	268.0371		0.33
24	Isorhamnetin O-Dhex-Dhex	15.7	267/349	607.1666	C_28_H_32_O_15_	299.058	284	0.15
25	Quercetin O-(Hex-Ac)	15.9	267/340	505.0969	C_23_H_22_O_13_	300.0727		0.24
26	Rosmarinic acid	16.1	218/287/329	359.0781	C_18_H_16_O_8_	161.023	197/179	5.95
27	Kaempferol O-glucuronide	27.2		461.0727	C_21_H_18_O_12_	285.0406	137	0.23
28	3-Dihydrocaffeoyl-4-caffeoyl quinic acid	16.4	215,325	517.1354	C_25_H_26_O_12_	355.1239	161/179/323	9.72
29	Kaempferol O-(Hex-Ac)	16.8		489.1036	C_23_H_22_O_12_	284.0325	255/227/327	0.72
30	Myricitrin glucuronide	18.5		519.1143	C_24_H_24_O_13_	314.0435	269/243/357	0.76
31	Apigenin O-(Hex-Ac)	18.8		473.1093	C_23_H_22_O_11_	268.0378		0.73
32	Biochanin A-hexurunosyl-hexurunosyl	19.5		637.1774	C_29_H_34_O_16_	283.0618		0.65
33	Apigenin O-(Hex-Ac)	20.1		473.1095	C_23_H_22_O_11_	268.0382		1.57

**Table 4:** Contents of the main bioactive compounds identified in the Mix2 analyzed in negative-ionization mode

No.	Compound	RT (min)	UV	*m/z* [M-H]^−^	Formula	MS^2^ main ion	MS^2^ fragments	mg/g DM
1	3-O-Caffeoylquinic acid	4.7	215/325	353.0880	C_16_H_18_O_9_	191.0553	179/161/135	6.91
2	2-O-Caffeoylhydroxycitric acid	5.2	215/324	369.0464	C_15_H_14_O_11_	189.003	127/207/179	0.78
3	1-O-Feruloylglucose	5.7	215/324	355.1046	C_16_H_20_O_9_	193.0498	149/0589/134	0.50
4	Caffeic acid	6.1		179.0342	C_9_H_8_O_4_	135.0436		1.09
5	Caffeoylmalic acid	6.2	250/324	295.046	C_13_H_12_O_8_	179.0336	133/207	1.27
6	2-O-Feruloylhydroxycitric acid	8.2	215/324	383.0623	C_16_H_16_O_11_	189.0039	191/337/127	3.64
7	N-Malonyl-D-phenylalanine	8.3		250.0723	C_12_H_13_NO_5_	165.055	207	1.88
8	Astilbin	9.2		449.1089	C_21_H_22_O_11_	287.0561	259/243	1.07
9	Nigellicine	9.4		245.0934	C_13_H_14_N_2_O_3_	203.0829		0.69
10	Quercetin O-Pen-Hex	10.1	255/352	595.1298	C_26_H_28_O_16_	300.0273		0.48
11	Isoquercitrin O-Dhex	10.2	252/351	609.1459	C_27_H_30_O_16_	300.0279		0.54
12	Quercetin O-Hex	10.5	252/351	463.0874	C_21_H_20_O_12_	301.0366	141/151	2.25
13	Typhaneoside	10.8	255/354	769.2207	C_34_H_42_O_20_	314.0436	595	2.71
14	Rutin	11.0	255/352	609.1451	C_27_H_30_O_16_	300.0275		5.73
15	6-Hydroxykaempferol 7-glucoside isoquercitrin	11.5	264/341	463.089	C_21_H_20_O_12_	300.0284		1.64
16	Quercetin-O-glucuronide	12.2	255/352	477.0681	C_21_H_18_O_13_	301.0359		0.85
17	Kaempferol O-Hex	12.3	265/343	447.0937	C_21_H_20_O_11_	285.0407		1.0
18	Sorhamnetin 3-O-Dhex-Hex	12.9		623.1631	C_28_H_33_O_16_	314.0436	461/161	0.25
19	Patuletin O-Hex	13.0	256/351	493.099	C_22_H_22_O_13_	331.0469	447/285/151	0.41
20	3,5-Dicaffeoylquinic acid	13.2	215/325	515.1208	C_25_H_24_O_12_	353.0885	173/179/191	0.92
21	Luteolin-O-Hex-Dhex	13.9	265/343	593.1518	C_27_H_30_O_15_	285.0409		1.43
22	Calendoflavoside	14.1	351/000	623.1627	C_28_H_33_O_16_	315.0515	269	0.71
23	3,4-Dicaffeoylquinic acid	14.5	215/326	515.1208	C_25_H_24_O_12_	353.0885	173/179/191	0.44
24	Apigenin O-Hex	14.7	266/339	431.0994	C_21_H_20_O_10_	268.0382		2.82
25	Isorhamnetin-O-Hex	14.9	267/338	447.0928	C_21_H_20_O_11_	284.0332	300/0279/327/255	1.11
26	1,5-Dicaffeoylquinic acid	15.2	214/325	515.1206	C_25_H_24_O_12_	353.0885	173/179/191	6.18
27	Isorhamnetin O-(Hex-Ac)	15.3	260/000	519.1141	C_24_H_24_O_13_	314.0436	331/299	1.75
28	Quercetin O-(Hex-Ac)	15.4	255/355	505.0963	C_23_H_22_O_13_	300.0275	271	0.25
29	3-caffeoyl-4-dihydrocaffeoyl quinic acid	15.9	215/325	517.1358	C_25_H_26_O_12_	323.0779	193/161/179/149/221	0.49
30	Apigenin O-(Hex-Ac)	16.5	265/325	473.1101	C_23_H_22_O_11_	268.0385		1.94

### EHT, LDT and parasitological status of lambs

**Fig. 1:**
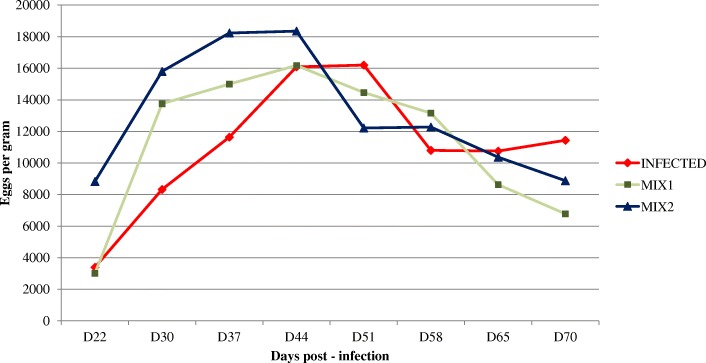
Mean fecal egg counts for the groups of lambs infected with *Haemonchus contortus*

In vitro anthelmintic activity of the methanolic and aqueous herbal mixtures extracts are presented in Table 5. Both mixtures generally affected hatching and development within the range of the test concentrations. The Mix1 and Mix2 results did not differ significantly in either in vitro test. The methanolic extracts, however, had a significantly stronger effect than the aqueous extracts in both mixtures and tests. The patterns of egg shedding for I, I + Mix1 and I + Mix2 are shown in Fig. 1. Data from D44 were statistically compared and used to determine the reduction in egg output for I + Mix1 and I + Mix2 relative to I. Mean fecal eggs per gram (EPGs) for all groups increased until D44. The EPGs in the lambs treated with Mix1 and Mix2 decreased from D44 until the end of the experiment. Eggs per gram for I similarly decreased, but only to D58 and then remained stable. The egg-output data indicated that mean EPG decreased between D44 and D70 for I + Mix1 and I + Mix2, by 58.1 and 51.6%, respectively. EPGs after D58 were always lower for I + Mix1 and I + Mix2 than I. Eggs per gram on D70 was significantly lower for I + Mix1 than I (*P <* 0.05). Mean body weights and live-weight gains did not differ significantly (*P >* 0.05) between the experimental groups. The necropsy on D70 found a significant decrease at abomasal worm count for I + Mix1 compared to I (Table 5).

**Table 5:** In vitro and in vivo anthelmintic activity against *Haemonchus contortus*

Part A: In vitro	Concentration (μg/mL)		
Egg hatch test ED_50_ ± SD	Methanolic extract	Aqueous extract	
Mix1	539.2 ± 174.4	940.0 ± 73.0**	
Mix2	486.9 ± 228.9	880.0 ± 67.8**	
Larval development test LD_50_ ± SD			
Mix1	266.6 ± 107.2	922.7 ± 232.1**	
Mix2	826.4 ± 370.5	1256.5 ± 465.0**	
Part B: In vivo	Numbers of adult worms		
Mean number of adult worms ± SD	Infected animals	Infected animals with Mix1	Infected animals with Mix2
	1523 ± 187	1113 ± 274*	1327 ± 351

In vitro activity: data are means ± SDs of three independent assays with duplicate samples. Ovicidal and larvicidal activities are expressed as the concentration (μg/mL) of a median lethal dose (ED_50_ and LD_50_, respectively; the concentration of a methanolic or aqueous extract that prevents 50% of the eggs from hatching or larvae from developing to the infective L3 stage) using the egg hatch test or larval development test

In vivo activity: *Reduction of adult worms relative to infected animals 70 days after infection (*P <* 0.05)

**Means within a row are significantly different at *P <* 0.01

### Effect on inflammatory, antioxidant and mineral parameters

Mean serum IgG concentrations were influenced by treatment and the treatment × time interaction (*P <* 0.001) and differed significantly (*P <* 0.01 and 0.001, respectively) for I vs. I + Mix1 by D22, D51 and D70 and for I vs. I + Mix2 by D22 (Table 6). Mean serum calprotectin concentrations were influenced by treatment, time and the treatment × time interaction (*P <* 0.001). Calprotectin concentrations for I + Mix2 ranged from 1.833 to 4.207 ng/mL and differed significantly for I vs. I + Mix2 by D51 and D70 (*P* < 0.001 and 0.01, respectively).

**Table 6:** Inflammatory responses of the experimental lambs

Parameter	Day	C	I	I + Mix1	I + Mix2	SD	Significance of effect				
							Treatment	I vs. I + Mix1	I vs. I + Mix2	Time	Treatment × time
IgG	22	0.073	0.321	0.544	0.525	0.0713	**	**	*	NS	**
(mg/mL)	37	0.074	0.352	0.425	0.482	0.0858		NS	NS		
	51	0.115	0.506	0.314	0.418	0.0931		**	NS		
	70	0.091	0.484	0.244	0.351	0.0566		**	NS		
Calpro	22	5.290	4.820	4.860	4.207	1.2650	**	NS	NS	**	**
(ng/mL)	37	3.505	1.900	2.997	2.777	0.9263		NS	NS		
	51	5.050	4.178	4.642	1.833	0.9462		NS	**		
	70	3.665	4.877	5.882	2.178	1.3114		NS	**		

*C* control uninfected animals, *I* infected animals, *I + Mix1* infected animals with Mix1, *I + Mix2* infected animals with Mix2, *Calpro* calprotectin, *N*S not significant

* *P <* 0.01, ** *P <* 0.001

Serum total antioxidant capacity (TAC), superoxide dismutase activity (SOD) and activity of blood glutathione peroxidase (GPx) (Table 7) were influenced by treatment (*P <* 0.001), and SOD and GPx activities were influenced by time (*P <* 0.05 and 0.001, respectively). Serum Zn concentration was influenced by treatment (*P <* 0.05) and time (*P <* 0.001) (Table 8).

**Table 7:** Antioxidant status in the experimental lambs

Parameter	Day	C	I	I + Mix1	I + Mix2	SD	Significance of effect				
							Treatment	I vs. I + Mix1	I vs. I + Mix2	Time	Treatment × time
TAC	22	0.552	0.537	0.538	0.543	0.055	***	NS	NS	NS	NS
(mmol/L)	37	0.593	0.508	0.562	0.568	0.060		NS	NS		
	51	0.667	0.502	0.517	0.535	0.102		NS	NS		
	70	0.560	0.458	0.550	0.548	0.075		NS	NS		
SOD	22	2020	3139	2705	3048	731.9	***	NS	NS	*	NS
(U/g Hb)	37	2566	3572	3510	3768	1271		NS	NS		
	51	2288	3592	3510	4206	1002		NS	NS		
	70	2865	3859	3895	3764	974.4		NS	NS		
GPx	22	0.114	0.109	0.108	0.101	0.038	***	NS	NS	***	NS
(U/mL)	37	0.101	0.052	0.064	0.055	0.030		NS	NS		
	51	0.091	0.075	0.051	0.053	0.022		NS	NS		
	70	0.092	0.076	0.044	0.055	0.030		NS	NS		

*TAC* total antioxidant capacity of serum, *SOD* superoxide dismutase activity in erythrocytes, *GPx* glutathione peroxidase activity in serum, *C* control uninfected animals, *I* infected animals, *I + Mix1* infected animals with Mix1, *I + Mix2* infected animals with Mix2, *NS* not significant

* *P <* 0.05, *** *P <* 0.001

**Table 8:** Mineral status in the sera of the experimental lambs

Element	Day	C	I	I + Mix1	I + Mix2	SD	Significance of effect				
							Treatment	I vs. I + Mix1	I vs. I + Mix2	Time	Treatment × time
Zn (mg/L)	22	0.476	0.462	0.567	0.591	0.07	*	NS	NS	***	NS
	37	0.548	0.477	0.512	0.519	0.03		NS	NS		
	51	0.590	0.512	0.542	0.582	0.04		NS	NS		
	70	0.687	0.590	0.548	0.682	0.07		NS	NS		
Fe (mg/L)	22	1.317	0.510	1.089	0.789	0.35	***	*	NS	***	*
	37	1.575	0.724	0.975	0.935	0.37		NS	NS		
	51	2.120	0.998	1.180	1.536	0.49		NS	NS		
	70	2.188	0.618	1.665	1.585	0.66		***	***		
Cu (mg/L)	22	0.774	0.651	0.834	0.841	0.09	NS	**	**	***	NS
	37	0.773	0.696	0.724	0.732	0.03		NS	NS		
	51	0.817	0.730	0.810	0.795	0.04		NS	NS		
	70	0.835	0.876	0.810	0.873	0.03		NS	NS		

*C* control uninfected animals, *I* infected animals, *I + Mix1* infected animals with Mix1, *I + Mix2* infected animals with Mix2, *NS* not significant

* *P <* 0.05, ** *P <* 0.01, *** *P <* 0.001

Serum Fe concentration was influenced by treatment (*P <* 0.001), time (*P <* 0.001) and the treatment × time interaction (*P <* 0.05). Serum Zn concentration differed significantly for I vs. I + Mix1 by D22 (*P <* 0.05) and D70 (*P <* 0.001) and for I vs. I + Mix2 by D70 (*P <* 0.001). Serum Cu concentration was influenced by time (*P <* 0.001) and differed significantly for I vs. I + Mix1 and I vs. I + Mix2 by D22 (*P <* 0.01).

### Effect on ovine feces microbial community

**Fig. 2:**
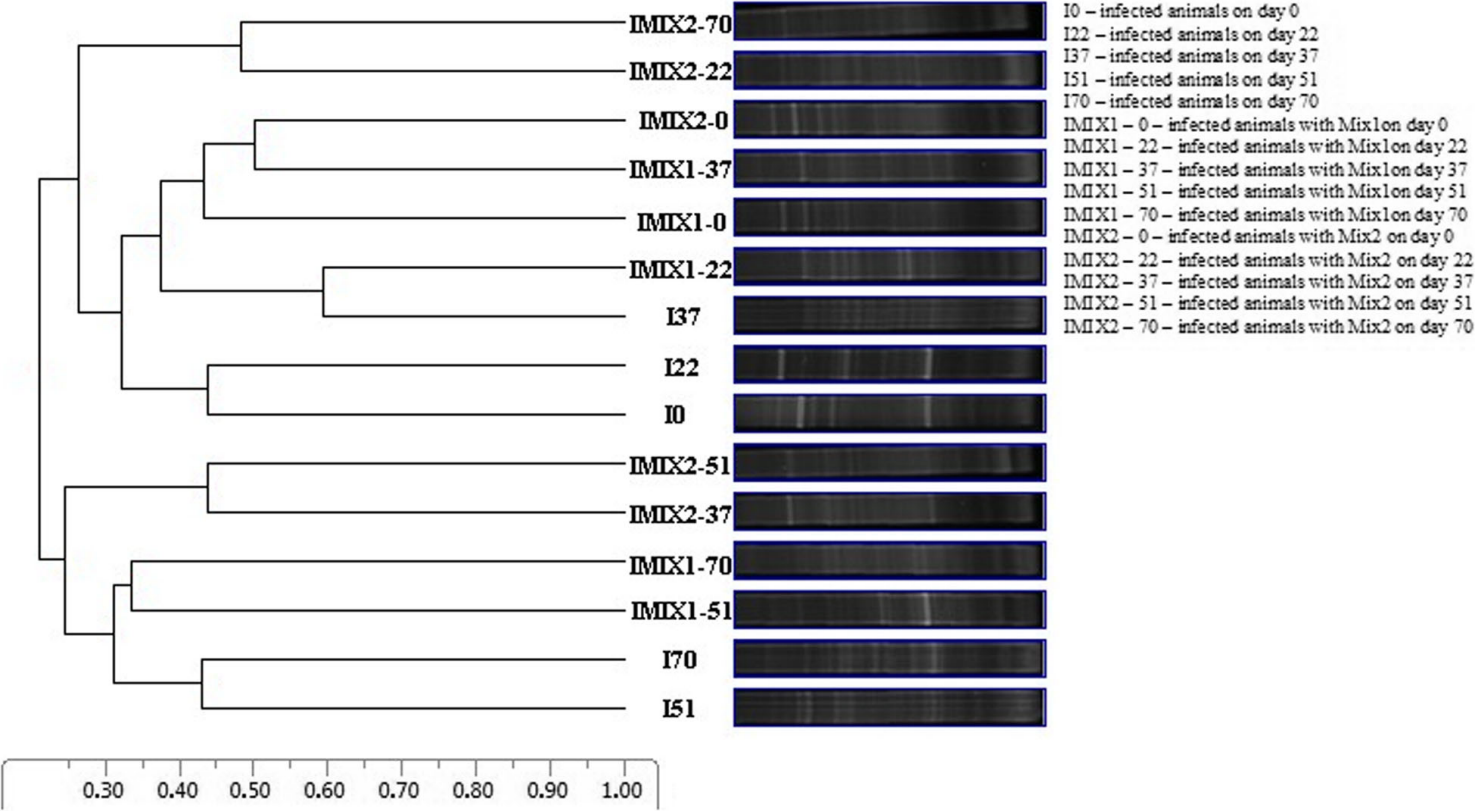
Unweighted pair group method with arithmetic mean algorithms (UPGMA) analysis of the DGGE profiles of the microbial communities from fecal samples of the lambs infected with *Haemonchus contortus* collected on days 0, 22, 37, 51 and 70 post-infection and treated with Mix1 and Mix 2

Denaturing gradient gel electrophoresis (DGGE) was used to analyze changes in the fecal microbial communities of the infected lambs induced by supplementation with Mix1 and Mix2 (Fig. 2). A comparison of the DGGE banding patterns indicated that the communities were only weakly affected by supplementation with Mix1 and Mix2. Most of the samples had similar banding patterns, with similarity coefficients ranging from 0.20 to 0.58. The cluster analyses (UPGMA) did not identify any herbal-mixture or animal-dependent clustering. None of the herbal treatments significantly affected the eubacterial microflora of the lambs infected with *H. contortus.*

## Discussion

The use of medicinal herbs containing bioactive compounds with important biological activities for preventing and treating GIN infections has its origin in traditional herbal medicine. Phenolic acids and flavonoids were the main bioactive compounds identified in both Mix1 and Mix2, but Mix1 contained more of these compounds than Mix2. Phenolic acids and flavonoids mitigate diseases associated with oxidative stress [25] and had excellent antioxidant activity in both our in vitro and in vivo experiments. Their anthelmintic activities also provide a potential option for treating nematode infections [26]. The phenolic acids 1,5-dicaffeoylquinic and 3-O-caffeoylquinic identified in both Mix1 (15.37 and 11.31 mg/g, respectively) and Mix2 (6.18 and 6.91 mg/g, respectively) possess antibacterial and anthelmintic activities [27]. Rosmarinic acid has well-known anti-inflammatory and antioxidant biological activities with beneficial health-promoting effects [28]. Its contents in Mix1 was 5.95 mg/g, two-fold higher than in a previously studied herbal mixture [23]. The presence of flavonol glycosides such as kaempferol, quercetin and myricetin contributes to the anthelmintic property of most browsed herbal species [29]. The herbal species in our study contained kaempferol (0.3–9.0 mg/g) and quercetin (0.5–7.7 mg/g), but the amount of myricetin was approximately three-fold higher in Mix1 than in another study [29]. Tannins and flavonoid glycosides may have similar mechanisms of action, because their chemical structures are similar [30]. All three groups of bioactive compounds also have antioxidant and anti-inflammatory properties [31, 32]. Another flavonoid with antioxidant activity, rutin, was also present in both Mix1 (2.96 mg/g) and Mix2 (5.73 mg/g), within the range of 2.3–10 mg/g reported for other herbal extract [33]. The flavonoid luteolin, with antioxidant and anti-inflammatory properties [34], was the most abundant compound in both Mix1 (Luteolin O-diglucuronide - 0.26 mg/g, Table 3) and Mix2 (Luteolin-O-Hex-Dhex - 1.43 mg/g, Table 4). Luteolin and quercetin can inhibit the larval development [35, 36].

The ultra-high-resolution mass spectrometry (UHRMS) analysis of the bioactive compounds in Mix1 and Mix2 also identified protoberberine-type alkaloids. Alkaloids in herbal species are commonly poisonous [37], but protoberberine alkaloids have a large variety of biological and pharmacological [38] and anthelmintic [39, 40] activities. The anthelmintic activity of herbs has sometimes been ascribed to alkaloids, which can interfere with DNA synthesis in parasites [41]. Several examples indicated protoberberine type alkaloids as valuable substances with anti-parasitic activities. Among the alkaloids tested to find new anthelmintics against parasites living in host tissues, allocryptopine (protoberberine type alkaloid) showed significant nematocidal activity against the larvae of dog roundworm, *Toxocara canis* with low cytotoxicity, and was proposed as a potentially effective anthelmintic [42]. *Fumaria indica* closely related to *Fumaria officinalis* (presented in both Mix1 and Mix2) is used as an anthelmintic in the traditional veterinary practices in Pakistan. The main alkaloids identified in this species responsible for those activities are protopine, fumarizine, papraine, papracine as well as flavonoids, glycosides, tannins and saponins [43]. In both herb mixtures was similar content of protoberberine-type alkaloids. Therefore it seems that better in vivo anthelmintic potential of Mix1 was probably mediated by content of phenolic acids and flavonoids. This is consistent with recent results reported limited anthelmintic potential of some alkaloids [44]. However, potent alkaloids probably act as antagonist of parasitic cholinergic receptors and lead to the development of novel drugs or may be used in combination with current anthelmintics to improve their efficacy [44].

The use of medicinal herbs containing bioactive compounds as an alternative treatment to chemical drugs is one approach that could reduce the development of resistance to antiparasitic drugs. One of our objectives was therefore to evaluate and compare the in vitro ovicidal and larvicidal efficacies of the aqueous and methanolic extracts of two herb mixtures against *H. contortus*. We previously evaluated the in vitro anthelmintic effects of aqueous and methanolic extracts from 13 species of medicinal herbs [45]. The results indicated that most herbs had a minimal effect on the hatching and larval development of *H. contortus* at the highest concentration used in the EHT and LDT. However, herbal extracts of four herbs (i.e., *Artemisia absinthium*, *Matricaria chamomilla*, *Fumaria officinalis* and *Malva sylvestris*) had strong anthelmintic activities in vitro [45]. We also previously showed that the wider spectrum of aqueous extracts from herbs (i.e., *Althaea officinalis*, *A. absinthium, M. chamomilla, F. officinalis* and *M. sylvestris*) exhibited stronger ovicidal activity and larvicidal activity in comparison to methanolic extracts of herbs; however methanolic extract of *A. absinthium* was the most efficient in both EHT and LDT [45]. These results suggest that herbal mixtures should be enriched with herbs with the highest anthelmintic potencies. Previous in vitro studies [46, 47] have only addressed the effects of individual herbal extracts on *H. contortus*. Our study is the first to monitor the ovicidal and larvicidal effects of herbal mixtures. The relatively high values (i.e., weak effects of extracts) in our in vitro tests suggested that the herbal mixtures could have an indirect antiparasitic effect in vivo and may promote the resistance of the host to parasitic infection only in the longer term. The effects were significantly stronger for the methanolic than the aqueous extracts in both the in vitro tests, suggesting a higher potency of the methanolic extracts, in accordance with some previous studies [48, 49] when aqueous and methanolic extracts did not produce the same anthelmintic response.

The egg outputs of our three parasitized groups (I, I + Mix1 and I + Mix2) indicated that egg reduction was largest in the I + Mix1 lambs. Egg production by *H. contortus* females has been reported to remain high to D50 post-inoculation and then to decrease [50]. We found the same pattern, where mean EPGs peaked from D44 to D51. The rapid reduction in egg excretion in the treated groups after D44, however, may have been due to the beneficial effects of the herbal treatments, supported by the dissections where the number of adult *H. contortus* worms was significantly lower for I + Mix1. The chemical composition of both herbal mixtures had optimal nutritional value and digestibility. Some herbs in Mix2 have yet to be evaluated for their anthelmintic properties under experimental conditions. Our previous studies, however, found no adverse effects on the patterns of ruminal fermentation when the diet was substituted up to 10% by a mix of some of the medicinal herbs used in Mix1 and Mix2 [22, 51]. Mix1 and Mix2 did not significantly affect the body weights or live-weight gains of the infected lambs, in contrast to the previous results [22]. Meta-analysis reported significant negative effect of parasitism on production in 58.3% of the trials [52]. The level of IgG antibodies against *H. contortus* increased significantly after infection in each infected group. This activity was apparent throughout the experiment. The necropsy confirmed the significant reduction in worm burden in the group fed Mix1, which was probably due to an immune response in the infected lambs. Associations of serum IgG levels with GIN infection and resistance against GIN infection in sheep have been reported [53, 54]. The ability to resist GIN infection, however, depends on the development of a protective acquired immune response, although the level of immunity depends on age, nutritional status and host genotype [20]. Calprotectin is a non-specific serum marker of neutrophil activation that directly correlates with chemical mediators of intestinal inflammation and with macroscopic and microscopic signs of disease [55]. The serum levels of calprotectin in our study were influenced by the herbal treatments. The response of serum calprotectin, however, was inconsistent with previous results [22, 23]. Some infectious and inflammatory diseases increase the serum levels of calprotectin [56], but calprotectin is mainly an early biomarker of bacterial infections [57].

Infected lambs generally have a reduced ability to absorb nutrients from the gastrointestinal tract, leading to morbidity and occasional mortality. Our results indicated that nematode infection in lambs was associated with oxidative stress and affected the antioxidant status of the animals. Both herbal mixtures containing bioactive compounds had strong antioxidant properties but did not have a beneficial effect on the serum antioxidant parameters in the infected lambs. The antioxidant potential of the mixtures or their bioactive compounds may have been influenced by several factors in vivo, including gut absorption, metabolism, bioavailability and the presence or absence of co-antioxidants and ions of transition metals [25]. The significantly lower serum Zn and Fe levels of the infected lambs relative to the control suggest a disturbance in their metabolism caused by *H. contortus*. Both mixtures had high iron contents, so differences between unsupplemented infected lambs and infected lambs supplemented with a herbal mixture could influence the serum Fe status during treatment [58]. Some medicinal herbs from central Europe containing essential microelements have been reported to possess antioxidant capacity which is correlated with the content of total phenolic compounds [59].

Adding the herbal supplement to the diets of infected lambs did not affect the composition of the fecal eubacterial communities in a substrate-specific manner. *Haemonchus contortus* manipulates the ovine gastrointestinal microbiome, modifying the balance between host and gastric microbiota [60]. Denaturing gradient gel electrophoresis can evaluate just the most dominant members of the bacterial community accounting for at least 3% of total population. We can assume that the main constituents of fecal microbial community were not influenced by the treatments. The diversity of the gastrointestinal microbiome is also extremely high, and the microbiome of adult *H. contortus* worms and L3 larvae differ between abomasa and feces [61]. Finding supplements that influence parasites, bacteria and the host is therefore important for the long-term maintenance of sheep health.

## Conclusion

The Mix1 had better in vivo anthelmintic potential than Mix2, probably due to its higher contents of phenolic acids and flavonoids. The in vitro results demonstrated an anthelmintic effect of the mixtures on *H. contortus*, but the in vivo experiments indicated that this effect was not sufficient for the primary elimination of parasites. Mix1, however, may affect the host over the longer term, leading to a reduction in parasitic infection intensity in the host. Multidisciplinary approach to control infection is important and provides a more complex view without overestimating partial results.

## Methods

### Experimental design, nutrition and animal management

Animal use and experimental design were approved by the Ethics Committee of the Institute of Parasitology of the Slovak Academy of Sciences in accordance with the national legislation in Slovakia, Animal Welfare Act (No. 23/2009). Permission to collect samples and to carry out the experiment was granted by the participating sheep farmers. Twenty-four female lambs (Improved Valachian) 3–4 months of age with initial body weights of 11.7 ± 1.23 kg were housed in common stalls for 15 d to acclimatize to the feeding treatments, with free access to water. The lambs were obtained from a commercial farm (Agricultural farm, Kluknava, Slovakia) and were maintained in their productive system during the experiment. All parasite-free lambs were then randomly divided on their live-weight into four groups (six lambs/group, one stall/group): uninfected control animals (C), animals infected with *H. contortus* (I), infected animals supplemented with herbal mixture 1 (I + Mix1) and infected animals supplemented with herbal mixture 2 (I + Mix2). The number of animals used in the experiment was assigned following VICH GL13 guidelines proposed by European Medicines Agency. Lambs were infected orally with approximately 5000 third-stage (L3) larvae of the MHco1 strain of *H. contortus*, which is susceptible to all main classes of anthelmintics. The experimental period was 70 days (during summer), and the animals were housed on a sheep farm. Lambs were fed the Mix1 and Mix2 (100 g DM/d/animal) during the experimental period.

### Parasitological techniques

Aqueous and methanolic extracts were prepared from both mixtures for EHTs and LDTs as was previously described [45]. Ovicidal activity was expressed as the concentration (μg/mL) of median effective dose (ED_50_, the concentration of a methanolic or aqueous extract that prevented 50% of the eggs from hatching). Larvicidal activity was expressed as the concentration (μg/mL) of median lethal dose (LD_50_, the concentration of a methanolic or aqueous extract that prevented 50% of the larvae from developing to the infective L3 stage).

Fecal samples from lambs were collected on D0, D22, D30, D37, D44, D51, D58, D65 and D70 post-infection and stored at 5 °C until laboratory examination. The detection of strongylid eggs was performed as was previously described [62]. All animals were humanely killed on D70 (abattoir of the Centre of Biosciences of SAS, Institute of Animal Physiology, Košice, Slovakia, No. SK U 06018), and helminthological dissections were performed [23]. The carcasses of animals were sent at the Department of Pathological Anatomy and Pathological Physiology, University of Veterinary Medicine and Pharmacy in Košice in Slovak Republic.

### Chemical analysis and fermentation parameters of the dietary substrates

The dietary substrates were analyzed in triplicate by standard procedures [63, 64]. The chemical composition of the Mix1 and Mix2, respectively, was (mean values, g/kg DM): DM: 898 (895); NDF: 500 (460); ADF: 360 (350): CP: 160 (180); N: 26 (29); ash: 110 (110); IVDMD: 600 (670); (mg/kg DM): Zn: 32.4 (36.7); Fe: 380 (396); Cu: 11.3 (13.8). IVDMD and in vitro measurements (Table 2) followed the procedures [22]. Ciliated protists in the fermentation ruminal fluid were counted microscopically and identified [65].

### Analysis of bioactive compounds

Phenolic acids and flavonoids in the samples of Mix1 and Mix2 were analysed as was previously described [23]. For the analysis of the protoberberine-type alkaloids, the Mix1 and Mix2 samples were ground to a fine powder, and 7 g of each were extracted with 0.5 M H_2_SO_4_ in an ultrasonic bath at 25 °C for 20 min. This procedure was then repeated, and the filtrates were combined, adjusted to pH 9–10 using 1 M NaOH and separated using CHCl_3_. The organic layer was collected, evaporated to dryness under reduced pressure and then dissolved in 80% MeOH for further analysis. The bioactive compounds were analyzed by UHRMS on a Dionex UltiMate 3000RS system (Thermo Scientific, Darmstadt, Germany) with a charged aerosol detector connected to a high-resolution quadrupole time-of-flight mass spectrometer (Compact, Bruker Daltonik GmbH, Bremen, Germany) as was previously described [23]. The total content of protoberberine-type alkaloids was determined as a chelidonine (CAS 476–32-4) equivalent from calibration curves based on seven concentration points of chelidonine (from 200 to 1.2 μg/mL). Alkaloids were separated using the same chromatographic conditions as for the phenolic compounds, except the gradient was from 7 to 70% phase B in phase A over 20 min. All analyses were performed in triplicate.

### Antioxidant and mineral parameters

Activity of blood glutathione peroxidase (GPx) and total antioxidant capacity (TAC) of the sera were determined as previously described [22, 23]. Superoxide dismutase (SOD) activity in erythrocytes was analyzed in fresh blood using a commercial kit (RANSOD, Randox Laboratories, Ltd., London, UK). Enzymatic activity was evaluated at 37 °C and a wavelength of 505 nm using an UV-VIS Spectrophotometer (UV-2550, Shimadzu Co., Kyoto, Japan) and the results are expressed in units/g hemoglobin. The serum mineral content was determined by flame atomic absorption spectrometry in an air-acetylene flame, with deuterium background correction, using an AA-7000 atomic absorption spectrophotometer (Shimadzu Co., Kyoto, Japan) as was described [22, 23].

### Inflammatory response

*Haemonchus contortus* antigen (HcAg) was obtained from ca. 50,000 L3. The larvae were washed twice in phosphate buffered saline (PBS, pH 7.4), diluted to a volume 15 mL and homogenized in an ice-cold glass (Sonopuls ultrasonic homogenizer HD3100, Bandelin, Germany). The homogenate was centrifuged (Heraeus Megafuge 16R, Thermo Fisher Scientific, Waltham, USA) at 4500 *g* for 5 min at 4 °C, and the supernatant was concentrated in 3000 MWCO VIVASPIN tubes (Sartorius, Goettingen, Germany) at 4000 *g* for 100 min at 4 °C. The protein concentration of the larval antigen was measured using the Bradford protein assay (Bio-Rad Laboratories, Munchen, Germany). Anti-*H. contortus* antibodies in the sera of the experimental lambs were detected using the HcAg somatic antigen and an indirect enzyme-linked immunosorbent assay (ELISA). *Haemonchus contortus* antigen was diluted to 5 μg/mL in carbonate buffer (pH 9.6) and then bound to microtiter plates (Nunc, Thermo Fisher Scientific, Roskilde, Denmark) at 4 °C overnight. The wells were washed three times with 0.5% Tween 20 in PBS (pH 7.4, PBS-T), and non-specific bonds were blocked with PBS containing 0.5% skimmed milk after 1 h of incubation at room temperature. The wells were again washed three times with PBS-T, and the serum samples were diluted to 1:100 and incubated at 37 °C for 1 h. All samples were examined in duplicate. The wells were washed again as above and bound antibodies were detected by incubating at 37 °C for 1 h with horseradish peroxidase-conjugated rabbit anti-sheep IgG (Sigma-Aldrich, Hamburg, Germany) diluted to 1:10000. The wells were washed again as above and 0.05 mol/l of the substrate o-phenylene diamine (Sigma-Aldrich, Hamburg, Germany) in citrate buffer (pH 4.7) with 0.005% H_2_O_2_ was used to induce a color reaction. The reaction was stopped by 1 M H_2_SO_4_ after a 15-min incubation at room temperature in the dark. The optical density was measured at 492 nm (Multiskan Reader, Thermo Fisher Scientific, Vantaa, Finland). The concentration of serum calprotectin was determined using commercial sheep ELISA kits (MyBioSource, Inc., San Diego, USA).

### DNA isolation and PCR amplification

Total DNA was extracted from frozen fecal samples using a QIAamp DNA Stool Mini Kit (Qiagen, Hilden, Germany). Isolated DNA was used as a template for the PCR amplification of 16S rRNA gene fragments. All PCRs were performed in 50-μL volumes containing 1 μL of DNA, 1 × PCR buffer, 2 mmol/L MgCl_2_, 1 μL of 200 μmol/L each dNTP, 1.25 U of Platinum Taq DNA polymerase (Invitrogen, New York, USA) and 25 pmol each primer using a C1000™ Thermal Cycler (Bio-Rad Laboratories, New York, USA). Universal primers fD1 (5′-AGA GTT TGA TCC TGG CTC AG-3′) and rP2 (5′-ACG GCT ACC TTG TTA CGA CTT-3′) [66] were used to amplify a 1500-bp region of the 16S rRNA gene in the first round of PCR. PCR conditions were: 94 °C for 5 min and then 35 cycles of 94 °C for 1 min, 54 °C for 1 min and 72 °C for 1 min 30 s, followed by 72 °C for 5 min. The 16S rRNA gene fragments were subsequently used as a template for the second round of PCR using the specific bacterial primers GC-clamp-968f (5′-CGC CCG GGG CGC GCC CCG GGC GGG GCG GGG GCA CGG GGG GAA CGC GAA GAA CCT TAC-3′) and 1401r (5′-CGG TGT GTA CAA GAC CC-3′) [67]. The cycling conditions were 94 °C for 5 min; 9 cycles of 94 °C for 1 min, 45 °C for 1 min and 72 °C for 1 min; 14 cycles of 94 °C for 1 min, 60 °C for 1 min and 72 °C for 1 min and a final extension at 72 °C for 10 min. The PCR products were detected by electrophoresis on a 0.8–1% agarose gel containing ethidium bromide and were photographed using a Gel Logic 212 PRO imaging system (Carestream, New York, USA). The second-round PCR products were subjected to DGGE. Denaturing gradient gel electrophoresis was performed using the DCodeTM Universal Mutation Detection System (Bio-Rad Laboratories, Hercules, USA). The PCR products in a total volume of 45 μL were loaded onto an 8% (w/v) polyacrylamide gel (37.5:1 acrylamide:bis-acrylamide) in 1 × TAE (40 mM Tris, 20 mM acetate, 1 mM EDTA) containing a linear denaturing gradient ranging from 30 to 60% denaturant (100% denaturant solution consisted of 7 M urea and 40% formamide). The electrophoresis was run for 17 h at a constant voltage of 51 V and a temperature of 60 °C. The gel was then incubated for 20 min in ethidium bromide (0.5 μg/mL), rinsed for 20 min in distilled water and photographed with UV transillumination using a Gel Logic 212 Pro Imaging System (Carestream, New York, USA).

### Calculations and statistical analysis

Analyses of variance (ANOVAs) (GraphPad Prism, GraphPad Software, Inc., San Diego, USA) were used for analyzing the inflammatory responses, antioxidant and mineral statuses as repeated-measures mixed models representing the four animal groups (C, I, I + Mix1 and I + Mix2) and sampling days. Effects included in the model were treatment, time and their interaction. Differences between the infected group (I) and both treated groups (I + Mix1 and I + Mix2) were analyzed by a two-way ANOVA with a Bonferroni post hoc test. Student’s *t*-tests were applied to assess the differences between the arithmetic EPG means on different sampling days from D44 for I, I + Mix1 and I + Mix2 and between worm counts at dissection. All EHT and LDT data were analyzed by Student’s *t*-tests to assess the differences between the arithmetic means of ED_50_/LD_50_ for Mix1 and Mix2 and between the aqueous and methanolic extracts. A probit model of regression analysis was applied to the data to express a median lethal dose (ED_50_ and LD_50_). Differences were determined using Tukey’s multiple comparison post hoc test. The significance level for all tests was set at *P <* 0.05.

The gel images were processed using CLIQS 1D Pro software (Total Lab Ltd., Newcastle, UK). A dendrogram representing band-pattern similarities was constructed using the unweighted pair group method with arithmetic mean (UPGMA) algorithms. UPGMA uses a sequential clustering algorithm, in which local topological relationships are identified in order of similarity, and a phylogenetic tree is sequentially built.

## Data Availability

The data sets used and/or analyzed are available from the corresponding author on reasonable request.

## Abbreviations

ADF: Acidic-detergent fiber
ANOVA: analysis of variance
C: control
Calpro: calprotectin
Concentrate: commercial concentrate composed of barley, soybean meal, wheat bran, bicarbonate and mineral–vitamin premix
CP: Crude-protein
D: day of sample collection
DGGE: denaturing gradient gel electrophoresis
DM: dry matter
DMSO: Dimethyl sulfoxide
EHT: egg hatch test
EPG: eggs per gram
GIN: gastrointestinal nematode
GPx: glutathione peroxidase
HcAg: *Haemonchus contortus* antigen
I + Mix1: infected animals with herbal mixture 1
I + Mix2: infected animals with herbal mixture 2
I: infected animals
IgG: immunoglobulin G
IVDMD: in vitro dry DM digestibility
LDT: larval development test
MH: meadow hay
MS^2^: tandem mass spectrometry
N: nitrogen
NDF: neutral-detergent fiber
NS: not significant
SOD: superoxide dismutase activity in erythrocytes
TAC: total antioxidant capacity
UHRMS: ultra-high resolution mass spectrometry
UPGMA: unweighted pair group method with arithmetic mean algorithms
